# Population screening for colorectal cancer: the implications of an ageing population

**DOI:** 10.1038/sj.bjc.6604788

**Published:** 2008-11-25

**Authors:** D A L Macafee, M Waller, D K Whynes, S Moss, J H Scholefield

**Affiliations:** 1 Department of Surgery, Royal Victoria Infirmary, Newcastle-upon, Tyne NE1 4LP, UK; 2 Institute of Cancer Research, 15 Cotswold Rd, Sutton, Surrey, UK; 3 School of Economics, University of Nottingham, Nottingham NG7 2RD, UK; 4 Division of Gastrointestinal Surgery, Wolfson Digestive Diseases Centre, Queen's Medical Centre, Nottingham University Hospitals, Nottingham NG7 2UH, UK

**Keywords:** colorectal cancer, modelling, economics, life expectancy

## Abstract

Population screening for colorectal cancer (CRC) has recently commenced in the United Kingdom supported by the evidence of a number of randomised trials and pilot studies. Certain factors are known to influence screening cost-effectiveness (e.g. compliance), but it remains unclear whether an ageing population (i.e. demographic change) might also have an effect. The aim of this study was to simulate a population-based screening setting using a Markov model and assess the effect of increasing life expectancy on CRC screening cost-effectiveness. A Markov model was constructed that aimed, using a cohort simulation, to estimate the cost-effectiveness of CRC screening in an England and Wales population for two timescales: 2003 (early cohort) and 2033 (late cohort). Four model outcomes were calculated; screened and non-screened cohorts in 2003 and 2033. The screened cohort of men and women aged 60 years were offered biennial unhydrated faecal occult blood testing until the age of 69 years. Life expectancy was assumed to increase by 2.5 years per decade. There were 407 552 fewer people entering the model in the 2033 model due to a lower birth cohort, and population screening saw 30 345 fewer CRC-related deaths over the 50 years of the model. Screening the 2033 cohort cost £96 million with cost savings of £43 million in terms of detection and treatment and £28 million in palliative care costs. After 30 years of follow-up, the cost per life year saved was £1544. An identical screening programme in an early cohort (2003) saw a cost per life year saved of £1651. Population screening for CRC is costly but enables cost savings in certain areas and a considerable reduction in mortality from CRC. This Markov simulation suggests that the cost-effectiveness of population screening for CRC in the United Kingdom may actually be improved by rising life expectancies.

Population screening for colorectal cancer (CRC) has recently commenced in the United Kingdom supported by the evidence of a number of randomised trials and pilot studies (Faive *et al*, 1991; Mandel *et al*, 1993; Winawer *et al*, 1993; Kewenter *et al*, 1994; Hardcastle *et al*, 1996; Kronborg *et al*, 1996; Towler *et al*, 1998). Certain factors are known to influence screening cost-effectiveness (e.g., compliance and faecal occult blood testing (FOBT) sensitivity), but it remains unclear whether an ageing population (i.e. demographic change) might also have an effect.

Colorectal cancer is currently the second commonest cause of cancer death in western society and its incidence rises steeply from 60 years of age to a peak at 72 years. In the United Kingdom, average life expectancy is currently estimated to be 75 but is expected to rise to 82 by 2050. The number of people aged above 60 years is also predicted to rise to 16 million by 2040 (United Nations, 2000; Office of National Statistics, 2002). There also remains some debate about the rate of this life expectancy rise. Oeppen and Vaupel (2002) described life expectancy gains of ‘2.5 years per decade’ although most current government predictions are closer to 1 year per decade currently.

Mathematical modelling is increasingly used in health-care programmes to assist in decision making processes, generalise on trial-based results and extrapolate from intermediate to final end points (Eddy, 1985; Buxton *et al*, 1997). The aim of this study was to simulate a population-based screening setting using a Markov model and assess the effect of a rising elderly population on CRC screening cost-effectiveness.

## Materials and methods

**Figure 1:**
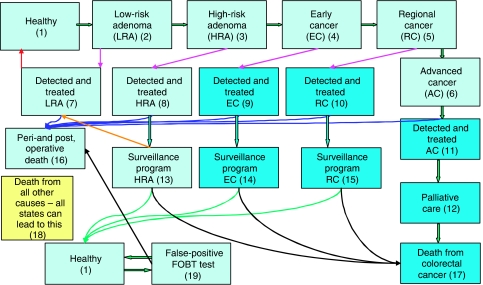
The Markov model constructed for colorectal cancer. The Markov model constructed for population screening for colorectal cancer using FOBT.

A Markov model was developed using STATA Version 8.0. Nineteen Markov disease states were constructed based on the well-established adenoma-carcinoma sequence (Leslie *et al*, 2002) (Figure 1). Dukes A and B cancers were classed as early, Dukes C cancers as regional and Dukes D cancers as advanced according to the Turnbull modification of Dukes staging (Dukes, 1932). Recurrent disease states were absorbed within the ‘detected and treated’ cancer states.

A Markov model characterises a disease and treatment process using a finite number of discrete, mutually exclusive health states. Progression through the model is based on transition matrices, which determine the probability of moving to a different health state. The Markov model assumes that the probability of moving to the next state is determined only by the present state and not by the path of past states. The model consisted of 19 health states and each cycle of the model corresponded to 1 additional year of follow-up. This model assumed that a patient could only move one state further in the disease progression in a given year (i.e. undetected low-risk adenoma to undetected high-risk adenoma).

The initial vectors and transition probabilities were established by a literature search of relevant articles, Pub Med, the Cochrane database, reference sorting and MESH headings with a search strategy of studies of CRC, average risk populations, published in the last decade, peer-reviewed, recognised governmental or research body websites and modelling studies of CRC (particularly Markov models).

A longitudinal design of the model was chosen and two cohorts were considered; an early cohort (commencing in 2003) and a late cohort (commencing in 2033). The cohorts analysed were assumed to be subject to age-specific mortality rates (all causes of death) in England and Wales in 2003, this being calculated using the 2003 Life Tables supplied by the UK Government Actuary Department (Government Actuary's Department, 2003). The early cohort, year 2003, consisted of 70 53 552 people aged 41–50 years. The cohort aged 11–20 years in 2003 were identified and survival data used to project the number surviving to 2033 (when they would be 50). This later cohort, consisting of 66 46 000 patients, was smaller than the 2003 cohort due to the lower birth rate in 1970 and 1980 decades. The actuary rates were then manipulated to consider a rise in life expectancy of both 1 and 2.5 years per decade. The World Health Organisation CRC mortality rates for the year 2002 were subtracted from the actuary rates to produce the standard all other cause rates used in the transition matrix (World Health Organisation, 2002).

The post-operative death rate was set at 3.5% for patients below 80 years, rising to 10% in those above 80 years of age (Smith *et al*, 2002). It was estimated that 1% of each cohort would be ineligible for screening at 50 years of age, due to conditions such as inflammatory bowel disease or CRC diagnosed earlier. Censoring occurred at the age of 100 years. Initially, the stage-specific annual death rates used in Frazier's model were used (0.002 – early cancer, 0.032 – regional cancer, 0.566 – advanced disease). After verification against the Nottingham screening results, these probabilities were raised so that the predicted number of deaths were more in line with those observed in the Nottingham trial. Frazier's CRC death probabilities were used in one of the sensitivity analyses of our model (Frazier *et al*, 2000b). Appendix 1 lists a number of assumptions underlying the model.

Appendix 2 illustrates the initial vectors, which are the probabilities of being in each disease state on the patient's fiftieth birthday (Hardcastle *et al*, 1996; Imperiale *et al*, 2000; Frazier *et al*, 2000b). The estimates of the prevalence of polyps and the proportion of polyps that were at high risk at 50 years of age were taken from Frazier *et al* (2000a). The proportions of the population with undiagnosed CRC were chosen to be consistent with a gradual decline through the states and sensible CRC incidence rates.

Each patient remained in a disease state for 1 year. The movement of patients at the end of each year was dependent on the transition probabilities assigned to that disease state. Although most transition probabilities remained constant, ‘all other cause death’, ‘post- and peri-operative death’ and ‘healthy to low-risk adenoma’ varied by patient age: the latter probabilities being kindly supplied by Dr Frazier (Frazier, 2002). Appendix 3 lists the transition probabilities for the main analysis (Frazier *et al*, 2000a; Alexander and Weller, 2003). The model considered a steady state with a cohort of 41- to 50-year-old people entering the model at 50 years of age, running a cohort simulation over a 50-year period. Appendices 4, 5 and 6 illustrate the Markov states and the movements between them (Safi and Beyer, 1993; Winawer *et al*, 1993; Hardcastle *et al*, 1996; Frazier *et al*, 2000b; Lund *et al*, 2001; Frazier, 2002; Nelson *et al*, 2002; Smith *et al*, 2002; Government Actuary's Department, 2003).

The sensitivity of FOBT for polyps and cancers was taken from the Cochrane review by Towler *et al* (2000). Compliance with CRC screening was altered depending on patient age (Farrands *et al*, 1984), and the model also considered the feature of screening detecting cancers that would have become incident in the absence of screening in a given year. The model also assumed that an increase in the number of screen-detected cancers and adenomas would lead to a reduction in the proportion of cancers progressing to a more advanced stage.

Initially, the sensitivity of FOBT for low- and high-risk adenomas was taken to be 10% (Frazier *et al*, 2000a). However, it became clear that a disproportionate number of low-risk adenomas were being detected and so the sensitivities were changed having reviewed data from the Nottingham trial and Towler Cochrane review of population screening (Hardcastle *et al*, 1986; Towler *et al*, 1998).

Population-based screening occurred every 2 years from age 60 to 69 years, which potentially saw five screening rounds and a long follow-up period (Scholefield *et al*, 2002). The time horizon for the study was 50 years in view of the rising life expectancy. For validation, the model results were compared with the Nottingham trial by increasing the number of screening rounds to six and considering the costs and outcomes up to 8 and 11 years of follow-up (Hardcastle *et al*, 1986; Scholefield *et al*, 2002). A screening period from 50 to 75 years of age was also considered that linked with the original Nottingham trial, and the results provide a useful picture of the implications of extending the number of screening rounds.

### Resources consumed

It was assumed that a patient with a low-risk adenoma would undergo one colonoscopy with 98% of patients returning to the healthy state in the subsequent year. Those with identified high-risk adenomas were assumed to require three colonoscopies; the original examination and two surveillance procedures over a 6-year period, before returning to the healthy state.

It was assumed that the primary operative intervention was similar for all Dukes disease stages. Guidance for post-endoscopic or -operative follow-up was taken from the British Society of Gastroenterology 2002 guidelines (British Society of Gastroenterology *et al*, 2002). Hospital-based surgical follow-up occurred for Dukes A to C cancers (over a 10-year period) and was modelled to involve four colonoscopies, eight clinic appointments, one CT and two ultrasound scans. Dukes D cancer patients either died in the year of diagnosis or were moved into the palliative care state the following year. The resources consumed by the detection and further treatment of recurrent disease were absorbed into the respective disease (detected and treated early or regional/palliative care) cancer disease states.

### Costs

Evidence for the costing of the model came almost exclusively from NHS reference costs (British Society of Gastroenterology *et al*, 2002; Department of Health, 2005). The individual costs of interventions are listed in Appendix 7. A treatment-level perspective of costs was taken for the analysis. The cost for hospital-based follow-up procedures was spread evenly over the period of surveillance.

Each year was considered separately with the number of persons in each disease state being multiplied by the cost of that state; the total cost of each cohort being derived from the sum of these yearly costs. Costs and outcomes were discounted at a 3.5% rate for the first 30 years of follow-up and 3% thereafter, with sensitivity analyses varying the rate to 0 and 10%. All costs were considered in sterling and updated to 2005 prices using the GDP deflator (HM Treasury, 2002). The costs of the test, postage, processing and subsequent investigation of positive tests were distributed across the disease states. The initial screening round occurred in the base year (year 0).

The number of detected pathologies, the number of CRC deaths, the cost of care and the effect of screening on CRC incidence and mortality were investigated and the cost of management examined. The four model outcomes, namely early and late-screened and non-screened cohorts, were then compared.

## Results

**Figure 2:**
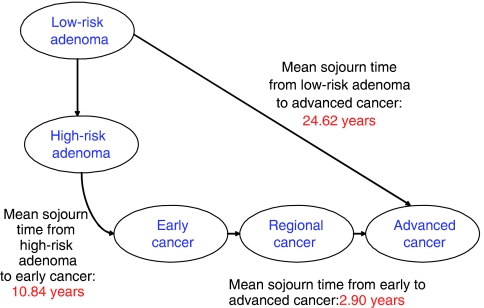
Mean sojourn time of the Markov model for progression through the adenoma-carcinoma sequence.

**Figure 3:**
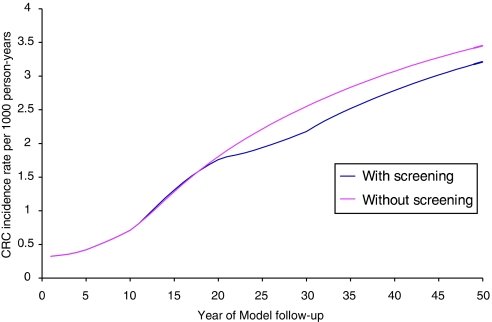
Colorectal cancer incidence rate in the late cohort with or without screening (60–69).

Owing to the lower birth rate in the later cohort (2033), there were 4 07 552 fewer people entering the model. As the adenoma-carcinoma sequence was the key process in the model, the sojourn time was examined and found to be clinically plausible (Figure 2). The reduction in the incidence rate of CRC was found in the screened cohort for both early and late cohorts; this fall first becoming apparent in the nineteenth year (cohort age 60–69 years), with clear divergence of the lines by 22 years (Figure 3). The distributions of all cancers detected in the screened group, by stage, were 43% (2 11 041) Dukes A and B, 33% (1 62 831) Dukes C and 24% (1 17 269) Dukes D in the early cohort.

Deaths from CRC reached 1 94 607 (65 983 discounted) at model cessation in the screened group of the later cohort. There were 30 345 fewer CRC deaths than the non-screened cohort. Overall, the relative rate of CRC mortality in the screened cohort at 30 years was 0.86 (Table 1).

Considering the screened late cohort, the trend of costs followed the marked rise in the number of low-risk adenomas detected by screening, with £5.8 million more spent on managing low-risk adenomas. High-risk adenoma management (detection, treatment and subsequent surveillance) cost £6.2 million more (Table 2). Early cancers proved to be the most costly stage to treat and survey, with the total costs reaching £556.3 million and £53.9 million, respectively. Overall, the cost of the screening programme was considerable, amounting to £96.2 million (Table 3). This represented 7% of the total cost of managing CRC in this 10-year cohort, the other major cost being primary treatment and detection, which cost £1204 million (83%).

Although screening led to a marked increase in high-risk adenoma detection (31 480 additional detected), there were 6495 fewer early stage cancers, 19 209 fewer regional cancers and 19 046 fewer advanced cancers. Owing to the reduced number of cancers treated, the cost of detecting, treating and caring for regional and advanced cancers reduced by £43 million and £30 million, respectively. Overall, screening the late cohort for CRC saw a cost rise by £26 million (Table 3).

The cost-effectiveness of biennial FOBT screening of the late cohort was found to be £1544 per life year saved after 30 years of follow-up. Considering an identical screening programme undertaken on the early cohort (2003), the cost per life year saved was £1651 (Table 4).

The cost per life year saved is initially very expensive due to the in-built costs of running a national screening programme. The overall cost of the programme would be expected to fall dramatically at the cessation of screening (at 69 years), whereas the benefits of the screening programme in terms of effect would continue. Overall, screening reduced the lifetime risk of CRC from 8.6 to 7.9%, with a relative rate reduction of 6% at 30 years. The relative rate reduction in CRC mortality was 14% over the same time period.

### Extending the number of screening rounds

The extended screening period (50–75) saw 1 69 246 CRC deaths, 25 361 fewer cancer deaths than the baseline model with fewer screening rounds. The relative rate of CRC mortality in the screened cohort at 30 years was 0.73. This improved detection rate and mortality reduction came at a cost of £141 million for screening, with an additional £99 million overall. The cost-effectiveness of screening from 50 to 75 was £1501 per life year saved, so proved to be similar in cost effectiveness to the 60–69 screening regiment. The lifetime risk of CRC was reduced from 8.6 to 6.3%, with a relative rate reduction of 27% at 30 years.

### Comparing life expectancy increases of 2.5 years *vs* 1 year per decade (screening from 50 to 75)

Compared with a 2.5-year per decade life expectancy rise, the 1-year rise saw fewer adenomas and cancers detected across the board. In the non-screening model in the late cohorts, the longer life expectancy saw an additional 51 631 early cancers, 44 377 regional cancers and 52 029 additional CRC deaths over the 50-year period. The average life expectancy dropped from 90 to 84 years. The cost of CRC care in all areas was less and, although totalling £1191.6 million, was £234.9 million less than for those with a higher life expectancy.

## Discussion

The cost per life year saved by screening was £1651 for the 2003 cohort and £1544 for the 2033 cohort at 30 years follow-up. Although screening of the cohort caused a relative cost increase in CRC care overall, it appears that the cost-effectiveness of FOBT screening does not deteriorate and may actually improve in a population with rising life expectancy.

A smaller increase in life expectancy of 1 year per decade saw an earlier age of death, fewer pathologies detected, less costs and a more favourable cost-effectiveness ratio. There was no difference in the relative risk reduction of CRC mortality.

Despite the lower birth rate in the later cohort and identical CRC incidence rates, it appears that the cost of CRC care without population screening would be increased compared with 30 years previously, which has resource implications for the UK health service (Table 3). The cost implications of such a trend included an additional £39 million for primary detection and treatment, £5.7 million for surveillance and £6 million for palliative care services. The overall cost of CRC care rose by 3.7% to £1426 million.

### Strengths and limitations

Although the sojourn time from high-risk adenoma to early cancer appeared to fit with current thinking (Figure 2), and model checking had corrected many small changes, it was clear that the mortality data (both for CRC and all cause) was deficient. Despite the changes made using the CRC rates from the Nottingham trial data, at 20 years follow-up, the number of CRC deaths was 2% above that of the trial with a CRC incidence 13% higher than the Nottingham screening population. These serve as a reminder that the results should be considered as projections of a situation up to the year 2083, with many potential factors affecting it.

This research commenced in 2002 at which time a UK-based national screening programme was still in the pilot stage with no government commitment for rolling it out. When the roll out was announced, Scotland elected to screen from 50 to 69 whereas England was initially restricted to 60–69. Reducing the number of screening rounds seems to slightly reduce the cost-effectiveness of screening.

Certain areas of Markov models remain unsatisfactory, one of which is the Markovian assumption. This states that the probability of leaving any particular state in the model is independent of the time spent in that state or the pathway followed to end up in that state. This means that the model has no memory, which is not an ideal situation when considered from a medical perspective (Drummond *et al*, 1997). Despite the weaknesses of Markov models, gaining an insight into this research question without such a mathematical model would be difficult. As only a 10-year cohort in England and Wales has been considered, it does not fully reflect all age groups in the United Kingdom, and rates would fluctuate further depending on the country in question. In a critical review of modelling systems for screening, Karnon *et al* (2007) highlighted the lack of methodological studies in this area and that there were no studies reporting direct empirical comparisons of alternative methodologies. As recommended in their review, we took separate disease stages (early, regional and advanced) and considered their post-diagnosis disease progression separately (Karnon *et al*, 2007). As with any model, there could be improvements and refinements. Future models could use gender-specific and more refined age-specific compliance rates for the uptake of screening. The most reliable source for this would probably be the national roll out study published by Alexander and Weller (2003). In terms of costs, the study by Trueman *et al* (2007) is probably the most comprehensive costing review of bowel cancer services in the literature to date.

This model is dependent on the reliability of three key papers; Frazier's model, the Nottingham FOBT trial (Hardcastle *et al*, 1996) and the UK pilot of population CRC screening (Alexander and Weller, 2003), but each of these has been recognised as reliable data at the time of publication. Frazier's study appears to be trusted and respected, being one of the more robust studies of its kind (Alexander and Weller, 2003). In an ageing population, the proportion of women will increase and the ethnic distribution may well change – neither of these areas were focussed on specifically in the paper and would be areas for future study.

Only direct hospital costs were considered here and the top-down approach taken for the NHS reference costs may underestimate the cost of treatment. Increasing use of adjuvant and palliative chemotherapy, intensive follow-up, metastatic cancer resections and increased resources for palliative care services will only raise the CRC costs further. Also, the initial costs of setting up a screening programme were not considered but are well recognised to be considerable (Garvican, 1998). These cost results could therefore be considered the minimum funds required (Dube *et al*, 1997; Memon and Beckingham, 2001; Ward *et al*, 2004).

Discounted costs and benefits were presented and the rates for costs were reduced from 3.5 to 3% after 30 years of follow-up as per standard economic evaluations (Drummond *et al*, 1997). Discounting benefits has a considerable effect on the number of CRCs detected and the overall mortality rate, and the whole process of discounting deaths and other outcomes remains debatable.

Using a cost per QALY measure as the cost-effectiveness outcome, rather than cost per life year gained, might make the results relevant to a wider audience and more easily comparable with non-cancer treatments. However, as the QALY coefficient in CRC screening is still considered to equal approximately one, it is likely that one life year gained continues to be comparable with one QALY in CRC screening (Whynes *et al*, 1994).

An alternative approach to sensitivity analysis could have been used, altering the chosen transition probabilities until a 10% change in the outcome measure was detected. Considering the transition probabilities for the movement through the adenoma-carcinoma sequence, the effect of a changing rate of transition has a considerable effect on the number of high-risk adenomas and cancers. Reducing the rate of low-risk to high-risk adenoma progression saw a 41–42% reduction in high-risk adenomas, early cancers and cumulative CRC deaths while also reducing total costs by 41% (£581.2 million less). Surprisingly, equally sized cohorts made little difference to costs (5% increase), whereas using the Frazier's cancer mortality rates increased costs by only 10%. Of all the cost changes to affect the results, altering the discount rate between 0 and 10% caused a huge change in results, with undiscounted costs (over the 50-year time horizon) being 177% higher. However, the costs are routinely discounted and so this figure is unrealistic in health economic terms.

Only the Minnesota randomised trials of FOBT screening have found a fall in CRC incidence, felt to be due to the high rate of colonoscopy with its annual screening programme. In this model, it took 10 years after screening started for any change in incidence to become evident but included a larger cohort. An overall gain in life expectancy of 2.4 months (screening from 50 to 75, 2.5 years per decade life expectancy gains) is higher than the National pilot figure of 14–16 days, with gains reducing to 1 month if lower life expectancy gains are used. Overall, the gains are not great and the World Cancer Report summarises the situation well: the screening benefit per person is small in terms of overall life expectancy (1–4 weeks), but the benefit is great for the 5% destined to have cancer (World Health Organisation, 2003).

## Conclusion

Population screening for CRC is costly but enables cost savings in certain areas and a considerable reduction in mortality from CRC. This Markov simulation suggests that the cost-effectiveness of population screening for CRC in the United Kingdom may actually be improved by rising life expectancies.

## Figures and Tables

**Table 1:** Number of subjects requiring care due to colorectal pathology in the late (2033) cohort in a screened population from 60 to 69 years of age

		**Detected and treated primary disease**				
**Years of follow-up**	**Discounted cumulative deaths from CRC *n* (undiscounted)**	**Low–risk adenoma *n* (undiscounted)**	**High-risk adenoma *n* (undiscounted)**	**Early cancer *n* (undiscounted)**	**Regional cancer *n* (undiscounted)**	**Advanced cancer *n* (undiscounted)**
*Late cohort – screened*						
1–20	16 901 (28 262)	35 625 (60 860)	44 643 (74 053)	30 814 (50 592)	21 074 (34 194)	13 835 (22 397)
21–40	52 869 (132 356)	28 265 (68 375)	51 490 (138 967)	41 853 (116 417)	32 224 (91 394)	23 239 (66 592)
41–50	65 983 (194 607)	2501 (10 995)	13 366 (58 848)	13 142 (57 881)	11 321 (49 869)	8686 (38 270)
Total	65 983 (194 607)	66 392 (140 230)	109 499 (271 868)	85 810 (224 891)	64 619 (175 458)	45 761 (127 260)
*Late cohort – not screened*						
Total	77 573 (224 952)	24 557 (56 427)	91 909 (240 388)	86 107 (231 386)	71 440 (194 667)	52 990 (146 306)

England and Wales population.

Life expectancy increasing at 2.5 years per decade.

2033 population of 6 646 000.

Screening with biennial unhydrated haemoccult faecal occult blood testing (FOBT).

**Table 2:** Costs of treating subjects with colorectal pathology in the late (2033) cohort in a screened population from 60 to 69 years of age

	**Detecting and treating primary disease**						**Cost of surveillance**			**Palliative care**
	**Low-risk adenoma**	**High-risk adenoma**	**Early cancer**	**Regional cancer**	**Advanced cancer**	**False positives**	**High-risk adenoma**	**Early cancer**	**Regional cancer**	
**Years of follow-up**	**£ million**	**£ million**	**£ million**	**£ million**	**£ million**	**£ million**	**£ million**	**£ million**	**£ million**	**£ million**
*Late cohort – screened*										
1–20	5.0	5.8	192.7	127.5	54.2	12.8	4.8	11.9	6.3	13.9
21–40	4.3	7.6	274.4	207.7	97.1	10.4	11.8	31.3	15.9	26.8
41–50	0.5	2.0	89.2	75.9	37.8	0.0	3.0	10.6	6.2	9.8
Total	9.8	15.4	556.3	411.2	189.1	23.2	19.7	53.9	28.3	50.4
*Late cohort – not screened*										
Total	4.0	12.8	557.1	454.2	219.1	0	16.1	53.4	31.3	78.4

England and Wales population, 2005 costs in pounds sterling discounted at 3.5% rate for the first 30 years of follow-up and 3% thereafter.

Life expectancy increasing at 2.5 years per decade.

2033 population of 6 646 000.

Screening with biennial unhydrated haemoccult faecal occult blood testing (FOBT).

**Table 3:** Comparing the costs of treating subjects with colorectal pathology with or without screening from 60 to 69 years of age: considering a 2003 (early) and 2033 (late) cohort

	**2003 (early cohort)**		**2033 (late cohort)**	
	**Non-screened**	**Screened**	**Non-screened**	**Screened**
	**Early cohort**	**Early cohort**	**Late cohort**	**Late cohort**
	**£ million (%)**	**£ million (%)**	**£ million (%)**	**£ million (%)**
Screening	—	100 (7)	—	96.2 (7)
Primary detection and treatment	1208.2 (88)	1174.0 (83)	1247.2 (87)	1203.8 (83)
Surveillance	95.2 (7)	96.8 (7)	100.9 (7)	101.9 (7)
Palliative care	72.4 (5)	46.4 (3)	78.4 (5)	50.4 (4)
Total cost of colorectal cancer care	1376	1417	1426	1452

2003 population of 7 053 552; 2033 population of 6 646 000.

England and Wales population, 2005 costs in pounds sterling discounted at 3.5% rate for the first 30 years of follow-up and 3% thereafter.

Life expectancy increasing at 2.5 years per decade.

**Table 4:** Comparing the cost per life year saved by screening a population from 60 to 69 years of age: 2003 and 2033 cohorts

**Screening group**	**Person years**	**Cost of care £ million**	**Cost per life year saved at 30 years** £
*Early cohort (2003)*			
Not screened	167 361 488	793.0	
			1650.8
Screened	167 424 446	896.9	
			
*Late cohort (2033)*			
Not screened	159 711 908	772.7	
			1544.2
Screened	159 775 486	888.0	

**Table A1:** Markov model: assumptions and screening model variables

**Assumptions**					
Dealing with an England and Wales population					
Life expectancy continues to improve at the present rate					
All subjects entering the model were assumed to be healthy or with undiagnosed adenoma or CRC					
Stage-specific survival remains the same					
Colorectal cancer remains an age-related disease					
Each subject remains in the Markov state for a full year					
The sojourn time from adenomatous polyp to cancer does not alter					
**Model variables**					
**Sensitivity of unhydrated faecal occult blood test for colorectal cancer**					
Colorectal cancer		33%	Towler *et al* (2000)		
High-risk adenoma		7.5%	Towler *et al* (2000 a		
Low-risk adenoma		2.5%	Towler *et al* (2000 a		
Age-specific compliance with screening		55%	50–64		Farrands *et al* (1984)
		48%	65–69		Farrands *et al* (1984)
		43%	70–74		Farrands *et al* (1984)
Screening unit costs	FOBT	£5.53	Processing of test		£0.21

Screening with biennial unhydrated haemoccult faecal occult blood testing (FOBT).

Sensitivity reduced, recognising that a higher proportion of high-risk to low-risk adenomas would be detected.

a Initially, 10% sensitivity for both low- and high-risk adenomas used, but unrealistic detection rate for FOBT.

**Table A2:** Initial vectors for the Markov model

**Disease states**	**Initial vector**	**References (date)**
Healthy	0.78875	One minus all other states
Low-risk adenoma	0.206	Frazier *et al* (2000a, 2000b)
High-risk adenoma	0.004	Frazier *et al* (2000a, 2000b)
Early cancer	0.001	Hardcastle *et al* (1996); Imperiale *et al* (2000)
Regional cancer	0.0002	Hardcastle *et al* (1996); Imperiale *et al* (2000)
Advanced cancer	0.00005	Hardcastle *et al* (1996); Imperiale *et al* (2000)
All other states	0	

A cohort of 50-year olds (population of England and Wales).

**Table A3:** Transition probabilities for the Markov model

**Variable**	**Value**	**References (date)**
Prevalence of polyps at age 50 years %	21	Frazier *et al* (2000a, 2000b)
Proportion of all polyps at age 50 years that are of high risk %	2	Frazier *et al* (2000a, 2000b)
		
*Annual transition probabilities*		
Normal epithelium to low-risk adenoma (age specific)		
50–54 years	0.005	Frazier (2002)
55–59 years	0.0065	Frazier (2002)
60–64 years	0.008	Frazier (2002)
Over 65 years	0.0095	Frazier (2002)
Low-risk to high-risk adenoma	0.02	Frazier *et al* (2000a, 2000b)
High-risk adenoma to early cancer	0.05	Frazier *et al* (2000a, 2000b)
Early to regional cancer	0.28	Frazier *et al* (2000a, 2000b)
Regional to advanced cancer	0.35	Alexander and Weller (2003) Estimate
		
*Probability of symptomatic presentation of colorectal cancer*		
Early cancer	0.25	Frazier *et al* (2000a, 2000b)
Regional cancer	0.45	Alexander and Weller (2003) Estimate
Advanced cancer	1	Frazier *et al* (2000a, 2000b)
		
*Annual colorectal cancer-specific mortality rates*		
Early cancer	0.0542	Hardcastle *et al* (1996); ACPGBI (2002)
Regional cancer	0.1677	Hardcastle *et al* (1996); ACPGBI (2002)
Advanced cancer	0.6469	Hardcastle *et al* (1996); ACPGBI (2002)
		
*Post/peri-operative death rates*		
Early cancer	0.057	ACPGBI (2002)
Regional cancer	0.069	ACPGBI (2002)
Advanced cancer	0.119	ACPGBI (2002)

**Table A4:** Markov states 1–6 and the links to other Markov states in the screening age range

**State number**	**Initial Markov state**	**Next Markov state (no.)**	**Transition probability**	**Reference (date)**
1	Healthy	Low-risk adenoma (2)	Age specific	Frazier (2002)
		Death from all other causes (16)	Age specific	Government Actuary's Department (2003)
		Remain (1)	^*^	
		False positive (19)	(0.03/2)^*^compliance	
2	Low-risk adenoma	High-risk adenoma (3)	0.02^*^(1−‘compliance’^*^sensitivity/2)	Frazier *et al* (2000a, 2000b)
		Detected and treated low-risk adenoma and discharged (7)	‘comp’^*^0.025/2+ (1−‘sens′^*^'comp’/2)^*^0.0009	Hardcastle *et al* (1996)
		Death from all other causes (16)	Age specific	Government Actuary's Department (2003)
		Remain (2)	^*^	
3	High-risk adenoma	Early cancer (4)	0.05^*^(1−‘sens’^*^sens/2)	Frazier *et al* (2000a, 2000b)
		Detected and treated high-risk adenoma (8)	‘comp’^*^0.075/2+ (1−‘comp’^*^sens/2)^*^0.019	Hardcastle *et al* (1996)
		Death from all other causes (16)	Age specific	Government Actuary's Department (2003)
		Remain (3)	^*^	
4	Early cancer	Regional cancer (5)	0.28^*^(1−‘comp’^*^sens/2)	Frazier *et al* (2000a, 2000b)
		Detected and treated early cancer (9)	‘comp’^*^0.33/2+ (1−‘comp’^*^sens/2)^*^0.18	Frazier *et al* (2000a, 2000b)
		Death from all other causes (16)	Age specific	Government Actuary's Department (2003)
		Remain (4)	^*^	
5	Regional cancer	Advanced cancer (6)	0.35^*^(1−‘comp’^*^'sens’/2)	Alexander and Weller (2003) Estimated
		Detected and treated regional cancer (10)	‘comp’^*^0.33/2+ (1−‘comp’^*^'sens’/2)^*^0.45	Alexander and Weller (2003) Estimated
		Death from all other causes (16)	Age specific	Government Actuary's Department (2003)
		Remain (5)	^*^	
6	Advanced cancer	Detected and treated advanced cancer (11)	^*^	
		Death from all other causes (16)	Age specific	Government Actuary's Department (2003)

Death from all other causes is an age-specific probability and so varies within the model.

^*^For each state, the transition probabilities for each state must add up to one.

2005 costs in pounds sterling discounted at 3.5% rate for the first 30 years of follow-up and 3% thereafter.

**Table A5:** Markov states 9–12 and the links to other Markov states in the screening age range

**State number**	**Initial Markov state**	**Next Markov state (no.)**	**Transition probability**	**Reference (date)**
7	D/T low-risk adenoma	Death from all other causes (16)	Age specific	Government Actuary's Department (2003)
		Healthy (1)	^*^	
		Peri/post-operative death (18)	0.0001	Nelson *et al* (2002)
		Remain (7)	0.019	Lund *et al* (2001)
8	D/T high-risk adenoma	Peri/post-operative death (18)	0.001	Nelson *et al* (2002
		Surveillance high-risk adenoma (13)	^*^	
		Death from all other causes (16)	Age specific	Government Actuary's Department (2003)
9	D/T early cancer	Peri/post-operative death (18)	0.057	ACPGBI (2002)
		Surveillance early cancer (14)	^*^	
		Death from all other causes (16)	Age specific	Government Actuary's Department (2003)
		Death from colorectal cancer (17)	0.0542	Hardcastle *et al* (1996); ACPGBI (2002)
10	D/T regional cancer	Peri/post-operative death (18)	0.069	ACPGBI (2002)
		Surveillance regional cancer (15)	^*^	
		Death from all other causes (16)	Age specific	Government Actuary's Department (2003)
		Death from colorectal cancer (17)	0.1677	Hardcastle *et al* (1996); ACPGBI (2002)
		Remain (10)	0.025	
11	D/T advanced cancer	Peri/post-operative death (18)	0.119	ACPGBI (2002)
		Palliative care (12)	^*^	
		Death from all other causes (16)	Age specific	Government Actuary's Department (2003)
		Death from colorectal cancer (17)	0.6469	Hardcastle *et al* (1996); ACPGBI (2002)
12	Palliative care	Death from all other causes (16)	Age specific	Government Actuary's Department (2003)
		Death from colorectal cancer (17)	0.6469	Hardcastle *et al* (1996); ACPGBI (2002)
		Remain (12)	^*^	

Death from all other causes is an age-specific probability and so varies within the model.^[2]^/T, detected and treated.

^*^For each state, the transition probabilities for each state must add up to one.

2005 costs in pounds sterling discounted at 3.5% rate for the first 30 years of follow-up and 3% thereafter.

**Table A6:** Markov states 13–19 and the links to other Markov states in the screening age range

**State number**	**Initial Markov state**	**Next Markov state (no.)**	**Transition probability**	**Reference (date)**
13	Surveillance	Healthy (1)	0.1667	
	High-risk adenoma	Detected and treated low-risk adenoma (7)	0.014	Winawer *et al* (1993); Lund (2002)
		Detected and treated high-risk adenoma (8)	0.023	Winawer *et al* (1993)
		Death from all other causes (16)	Age specific	Government Actuary's Department (2003)
		Remain (13)	^*^	
14	Surveillance	Healthy (1)	0.125	
	Early cancer	Death from all other causes (16)	Age specific	Government Actuary's Department (2003)
		Death from colorectal cancer (17)	0.0239	Hardcastle (2000)
		Remain (14)	^*^	
15	Surveillance	Healthy (1)	0.125	
	Regional cancer	Death from all other causes (16)	Age specific	Government Actuary's Department (2003)
		Death from colorectal cancer (17)	0.0649	Hardcastle (2000)
		Remain (15)	^*^	
16	Death from other cause	Permanent state	—	—
17	Death from colorectal cancer	Permanent state	—	—
18	Peri/post-operative death	Permanent state	—	—
19	False-positive tests	Healthy (1)	^*^	
		Death from all other causes (16)	Age specific	Government Actuary's Department (2003)
		Peri/post-operative death (18)	0.0001	

Death from all other causes is an age-specific probability and so varies within the model.

^*^For each state, the transition probabilities for each state must add up to one.

2005 costs in pounds sterling discounted at 3.5% rate for the first 30 years of follow-up and 3% thereafter.

**Table A7:** Cost of interventions in colorectal cancer care

	**Unit cost (£)**	**Sensitivity analysis**		
**Procedure**	**Base**	**Lowest**	**Highest**	**References (date)**
*Screening*				
Faecal occult blood test	5.53	4.43	6.64	Department of Health (2005) ^*^^,+^
Processing of test	0.21	0.17	0.25	Whynes (2002)^*^
				
*Investigation and intervention*				
Clinic appointment	97	85	113	Department of Health (2005) FUA 114
Computed tomography (CT) scan	86	63	101	Department of Health (2005) RBC6
Ultrasound of liver	67	51	89	Department of Health (2005) RBC2
Colonoscopy	133	122	223	Department of Health (2005) F35
Surgical resection and follow-up				
Early	4756.09	3804.87	5707.31	Alexander and Weller (2003) ^*^^,+^
Regional	4518.18	3614.54	5421.81	Alexander and Weller (2003) ^*^^,+^
Advanced	2378.05	1902.44	2853.66	Alexander and Weller (2003) ^*^^,+^
Post-operative chemotherapy	276	147	463	Department of Health (2005) X99COC
Palliative care	2909.21	2327.36	3491.05	Alexander and Weller (2003) ^*^^,+^

£ pounds sterling at 2005 costs.

^*^Updated to 2005 costs.

+Ranges of costs calculated as 20% above and below base rate.

Department of Health: NHS reference costs.

BSG: British Society of Gastroenterology Guidelines.

## Appendix 1

Table A1

## Appendix 2

Table A2

## Appendix 3

Table A3

## Appendix 4

Table A4

## Appendix 5

Table A5

## Appendix 6

Table A6

## Appendix 7

Table A7
